# Specific gut bacterial and fungal microbiota pattern in the first half of pregnancy is linked to the development of gestational *diabetes mellitus* in the cohort including obese women

**DOI:** 10.3389/fendo.2022.970825

**Published:** 2022-09-05

**Authors:** Marketa Vavreckova, Natalie Galanova, Martin Kostovcik, Ondrej Krystynik, Eliska Ivanovova, Radka Roubalova, Zuzana Jiraskova Zakostelska, David Friedecky, Jaroslava Friedecka, Martin Haluzik, David Karasek, Klara Kostovcikova

**Affiliations:** ^1^ Laboratory of Cellular and Molecular Immunology, Institute of Microbiology of the Czech Academy of Sciences, Prague, Czechia; ^2^ Laboratory of Fungal Genetics and Metabolism, Institute of Microbiology of the Czech Academy of Sciences, Prague, Czechia; ^3^ Third Department of Internal Medicine – Nephrology, Rheumatology and Endocrinology, University Hospital Olomouc, Olomouc, Czechia; ^4^ Laboratory for Inherited Metabolic Disorders, Department of Clinical Biochemistry, University Hospital Olomouc, and Faculty of Medicine and Dentistry, Palacky University Olomouc, Olomouc, Czechia; ^5^ Diabetes Centre, Institute for Clinical and Experimental Medicine, Prague, Czechia

**Keywords:** microbiome, mycobiome, early diagnosis, plasma metabolites, short-chain fatty acids, correlation

## Abstract

**Aims:**

Gestation is linked to changes in gut microbiota composition and function. Since gestational *diabetes mellitus* (GDM) can develop at any time of the pregnancy, we stratified the women into four groups according to the time and test used for the diagnosis. We focused on the gut microbiota pattern in early pregnancy to detect changes which could be linked to later GDM development.

**Methods:**

We collected stool samples from 104 pregnant women including obese individuals (first trimester body mass index median was 26.73). We divided the women into four groups according to routine screening of fasting plasma glucose (FPG) levels and oral glucose tolerance test (oGTT) in the first and third trimesters, respectively. We processed the stool samples for bacterial 16S rRNA and fungal ITS1 genes sequencing by Illumina MiSeq approach and correlated the gut microbiota composition with plasma short-chain fatty acid levels (SCFA).

**Results:**

We found that gut bacterial microbiota in the first trimester significantly differs among groups with different GDM onset based on unweighted UniFrac distances (p=0.003). Normoglycemic women had gut microbiota associated with higher abundance of family Prevotellaceae, and order Fusobacteriales, and genus *Sutterella*. Women diagnosed later during pregnancy either by FGP levels or by oGTT had higher abundances of genera *Enterococcus*, or *Erysipelotrichaceae UCG-003*, respectively. We observed significant enrichment of fungal genus *Mucor* in healthy pregnant women whereas *Candida* was more abundant in the group of pregnant women with impaired oGTT. Using correlation analysis, we found that *Holdemanella* negatively correlated with *Blautia* and *Candida* abundances and that *Escherichia*/*Shigella* abundance positively correlated and *Subdoligranulum* negatively correlated with plasma lipid levels. *Coprococcus*, *Akkermansia*, *Methanobrevibacter*, *Phascolarctobacterium* and *Alistipes* positively correlated with acetate, valerate, 2-hydroxybutyrate and 2-methylbutyrate levels, respectively, in women with GDM.

**Conclusions:**

We conclude that there are significant differences in the gut microbiota composition between pregnant women with and without GDM already at the early stage of pregnancy in our cohort that included also overweight and obese individuals. Specific microbial pattern associated with GDM development during early pregnancy and its correlation to plasma lipid or SCFA levels could help to identify women in higher risk of GDM development.

## Introduction

Gestational *diabetes mellitus* (GDM) is the most common medical complication of pregnancy that affects more than 14% of women worldwide (1). It is described as any degree of glucose intolerance that appears during pregnancy (2). GDM is associated with many health complications affecting woman and the offspring, including gestational hypertension, preeclampsia, or preterm birth and fetal macrosomia, hypoglycemia, respiratory distress syndrome or cardiomyopathy (3, 4). In addition, women with GDM have about 40% higher risk of developing type 2 *diabetes mellitus* (T2DM) in the next 10 – 15 years (5, 6). The offspring of women with GDM are in increased risk for developing diabetes and obesity as well (5, 7). Nevertheless, several studies have described that infants breastfed by women diagnosed with GDM may have reduced risk of obesity or T2DM development later in their life (8, 9). Factors transferred by milk from mother to offspring modulate its microbiome, immune system tuning or metabolic activity which are tightly associated with obesity or T2DM (10–12). Intrinsic and extrinsic factors accompanying the metabolic and immunological changes during pregnancy, especially increased insulin resistance, gestational weight gain, family history of diabetes, obesity and immune tolerance against the fetus and placenta, are the prerequisite for the development of GDM (13, 14). These changes are also associated with alterations in the energy metabolism of pregnant women. The beginning of pregnancy is strongly related with the storage of energy. However, in the third trimester, the energy metabolism pathways are activated, which results in the release of glucose and fatty acids into the bloodstream (15). As a consequence of these significant changes in the metabolism, predisposed pregnant women are prone to develop GDM. Based on the diagnostic criteria, two main subtypes of GDM may be distinguished. The first one is characterized by women with repeatedly increased fasting plasma glucose (FPG; FPG ≥ 5.1 mmol/l) while the second one is detected postprandially after oral glucose tolerance test (oGTT; plasma glucose ≥ 10.0 mmol/l at 1h and/or ≥ 8.5 mmol/l at 2h during oGTT). These two subtypes differ in their pathophysiological mechanisms and also in the severity of health complications associated with GDM (16). This means the earlier GDM develops the more severe complications it brings. Therefore, prompt diagnosis is crucial for early dietary intervention and mitigation of the consequences.

The composition and metabolic activity of the gut microbiota have been described as factors that can influence glucose metabolism. For instance, a specific gut microbiota pattern has been observed in subjects with obesity, prediabetes or T2DM (17–19). Moreover, microbial diversity and its function in the gut are altered during pregnancy. In the first trimester, the gut microbiome of a pregnant woman is mostly similar to a healthy non-pregnant woman, while in the third trimester, a high degree of dysbiosis is observed, especially in the decrease of short-chain fatty acids (SCFA)-producing bacteria and in the increase in Actinobacteria and Proteobacteria (20, 21). Although the relative abundance of the four dominant phyla (Firmicutes, Bacteroidetes, Proteobacteria and Actinobacteria) differs among mildly underweight pregnant women, pregnant women with normal body mass index (BMI), overweight, and obese pregnant women (22), any of these women can develop GDM. To date, few studies have focused on microbial or microbiota-associated metabolic changes in GDM development, and none of them have aimed at the differences in the gut microbiota composition among subtypes of GDM determined by FPG levels or oGTT.

The microbiota can also modify metabolic processes in the body through their metabolites, such as SCFA, branched-chain fatty acids or bile acids. SCFA are produced by gut microbiota through anaerobic fermentation of non-digestible carbohydrates. Major types of SCFA are acetate, propionate and butyrate that modulate energy metabolism and that are involved in the maintaining of glucose homeostasis (23). However, type and amount of SCFA depends on diet that affects the gut microbiota composition and function (24). Recently, the impaired insulin sensitivity in pregnancy and the development of GDM have been linked to diet as a source of substrates that are further processed by the gut microbiota, resulting in the formation of metabolites, such as SCFA (25, 26).

In our study, we focused on the early gut microbiota pattern in pregnant women in order to identify changes which could predict later GDM development. For this purpose, we sequenced gut bacterial and fungal microbiota in 104 pregnant women, representing a common Czech population of women with low, normal and high BMI. The women were divided into four subgroups according to their FPG levels and oGTT as follows: healthy pregnant women, pregnant women with impaired FPG in the first trimester, pregnant women with impaired FPG in the third trimester and pregnant women with impaired oGTT in the third trimester. Moreover, we correlated the microbiota changes with basic biochemical parameters and SCFA levels in plasma. Our data could help to determine early pregnancy microbial patterns that are associated with GDM development later during pregnancy and thus could help with its early detection.

## Subjects, materials and methods

### Study subjects and sampling

For this study, 104 pregnant women were enrolled during regular appointments at the Third Department of Internal Medicine – Nephrology, Rheumatology and Endocrinology, Olomouc University Hospital. The exclusion criteria for enrolment comprised of recent antibiotic treatment (at least three months before sampling) and a history of intestinal disease or major intestinal resection. Enrolled women were tested for GDM according to the recommendation of International Association of Diabetes and Pregnancy Study Groups (27). The detection and diagnosis of hyperglycemic disorders in pregnancy involves two phases. The first test is performed during an initial prenatal visit (usually in the first trimester) to reveal women with overt diabetes who have not been diagnosed before pregnancy. If the results are not sufficient for the diagnosis of overt diabetes but are abnormal (FPG ≥ 5.1 mmol/L but < 7.0 mmol/L), early GDM is suspected. Therefore, if overt diabetes is excluded, it is recommended to classify as GDM also the FPG values ≥ 5.1 mmol/L in early pregnancy. The second phase includes the 75g oGTT in 24th – 28th week of gestation in all women who had not previously been diagnosed with overt diabetes or GDM to detect GDM in this period. Clinical and biochemical parameters were collected in the first trimester of pregnancy from the patients’ registry and the SCFA levels were extracted from a publication by Ivanovova et al. (2021) which describes the same cohort of women with GDM (28). Samples of feces were collected at two time points during the first and the third trimesters of pregnancy. Samples were frozen within 5h after collection and stored at -20°C until the DNA extraction.

This study was approved by the Ethics Committee at Olomouc University Hospital (approval no. 120/17). An informed consent was obtained from all subjects before enrolment.

### DNA extraction from stool samples and sequencing

Total DNA was extracted using ZymoBIOMICS DNA Miniprep Kit (ZYMO Research, Irvine, CA, USA)according to the manufacture´s protocol with repeated bead-beating using FastPrep homogenizer (MP Biomedicals, Santa Ana, CA, USA). PCR targeting V3 and V4 regions of bacterial 16S was conducted using Kapa HiFi HotStart Ready mix (Roche, Penzberg, Germany) using 341F (5′-CCTACGGGNGGCWGCAG- 3′) and 806R (5′-GGACTACHVGGGTWTCTAAT- 3′) primers (Generi Biotech, Hradec Kralove, CZ). Cycling conditions consisted of initial denaturation (95°C, 4 min) followed by 30 cycles of denaturation (95°C, 30 s), annealing (55°C, 30 s), extension (72°C, 30 s) and final extension (72°C, 5 min). PCR targeting of fungal ITS1 region was performed also with Kapa Hifi HotStart Ready mix (Roche) using primers with barcodes ITS1-5.8Sfw (5′-AAGTTCAAAGAYTCGATGATTCAC-3′) and ITS1-5.8Srv (5′-AAGTTCAAAGAYTCGATGATTCAC-3′). Cycling conditions consisted of initial denaturation (95°C, 4 min) and 35 cycles of denaturation (95°C, 30 s), annealing (60°C, 30 s), extension (72°C, 30 s) and final extension (72°C, 5 min). PCR triplicates were pooled and purified by SequalPrep Normalization Plate Kit (Thermo Fisher Scientific, Waltham, MA, USA). Samples within library were pooled, concentrated (Eppendorf centrifugal vacuum concentrator), purified with DNA Clean&Concentrator kit (ZYMO Research)and sequencing adaptors were ligated using Kapa HyperPrep kit (Roche). Ligated libraries were quantified with KAPA Library Quantification Kit (Kapa Biosystems) and sequenced on MiSeq Illumina Platform using Miseq Reagent Kit v3 (Illumina) at The Genomics Core Facility, CEITEC (Brno, Czech Republic). Sequencing data were processed using QIIME version 1.9.1 (29). Raw reads were demultiplexed and quality filtered, allowing no N characters, a maximum of three consecutive low-quality base calls, a maximum unacceptable Phred quality of Q20, and a maximum of 1.5 barcode errors. Chimeric reads were detected and discarded using USEARCH algorithms (30). Fungal reads were in addition extracted for ITS1 region using ITSx package (31). Identification of representative sequences was done using RPD classifier (32) against bacterial GREENGENES database 13.8 (33) and fungal UNITE database 7.2 (UNITE Community (2017): UNITE QIIME release. Version 01.12.2017. UNITE Community. https://doi.org/10.15156/BIO/587481). Finally, OTU table was produced. The data are available in the Sequence Read Archive (SRA) https://www.ncbi.nlm.nih.gov/bioproject/PRJNA833950.

Briefly, for microbiota analysis, the number of observed OTUs (operational taxonomic unit) and Chao1, Shannon, Simpson and Faith Phylogenetic Diversity indexes were used to describe alpha diversity and Principle Coordinate Analysis (PCoA) based on weighted and unweighted UniFrac distance for bacteria and Bray-Curtis and Jaccard distance for fungi were used to characterize beta diversity. The permutational multivariate analysis of variance (PERMANOVA) was used for the determination of statistical differences among groups. Furthermore, Linear discriminant analysis effect size (LEfSe; RRID: SCR_014609) was used to determine the features discriminating communities in each group (29, 34). Functional potential of a bacterial metagenome was predicted by Phylogenetic Investigation of Communities by Reconstruction of Unobserved States (PICRUSt) tool, using the 16S rRNA amplicon data (35).

### Statistics

Data were analyzed using GraphPad Prism version 8.0.0 for Windows (GraphPad Software, San Diego, CA, USA; www.graphpad.com). Statistical differences between two groups were calculated by nonparametric Mann-Whitney U test. In the case of more groups, nonparametric Kruskal-Wallis test with Dunn´s *post-hoc* testing were used. Data were expressed as medians with first and third quartiles. Values of p < 0.05 were considered significantly different. Covariations of gut microbiota with other factors were calculated by Spearman´s correlation analysis with Bonferroni ´s adjustment for multiple comparisons.

## Results

### Clinical data and blood samples analyses

For basic group differentiation, we compared clinical and biochemical parameters of healthy pregnant women (HC) and pregnant women with diabetes who had impaired FPG in the first (GDM1) or in the third trimester (GDM2), and with impaired oGTT in the third trimester (GMD3; **Table 1**). We found a significant increase in body weight (GDM2 p<0.01; GDM3 p<0.05) and BMI (GDM1 p<0.01; GDM2 p<0.001; GDM3 p<0.01) in women with GDM compared to healthy pregnant women mainly due to obese women inclusion. The GDM2 and GDM3 groups showed significant increase in cholesterol (GDM2 p<0.001; GDM3 p<0.01), triglycerides (GDM2 p<0.001; GDM3 p<0.001), low-density lipoprotein (LDL; GMD2 p<0.01; GDM3 p<0.05), non-high-density lipoprotein (nonHDL; GDM2 p<0.001; GDM3 p<0.01) and 3-hydroxybutyrate (GDM p<0.05; GDM p<0.001) compared to healthy pregnant women. The highest FPG levels were determined in the GDM1 (p<0.001) and GDM2 women (p<0.001).

**Table 1:** First trimester clinical and biochemical data of healthy pregnant women and pregnant women with impaired FPG or oGTT.

Group [n]	HC (22)	GDM 1 (29)	GDM 2 (31)	GDM 3 (22)
Definition	Healthy pregnant women with normal FPG (FPG < 5.0 mmol/L)	Pregnant women with impaired FPG (FPG ≥ 5.1 mmol/L) in the first trimester	Pregnant women with impaired FPG (FPG ≥ 5.1 mmol/L) in the third trimester	Pregnant women with impaired oGTT in the third trimester
Age [y]	23 – 36 30 (28; 32)	21 – 46 31 (28;34)	24 – 44 32 (27;36)	25 – 40 32 (28;35)
Body height [cm]	158 – 179.5 172 (165.5; 176.5)	155 – 183 168 (162; 172)	151 – 177 166 (162; 172)	155 – 176 168 (163; 172)
Body weight [kg]	55 – 111 67.5 (62.3; 74.9)	52 – 126 78 (68; 84)	59 – 118 81.5 (70; 90)**	51 – 110 80 (68; 97)*
BMI [kg/m^2^]	19.55 – 38.41 23.5 (20.6; 25.7)	17.58 – 37.91 27.4 (24.4; 32.1)**	22.76 – 44.53 28.7 (24.8; 31.4)***	18.29 – 44.12 28.9 (24; 33.8)**
Obese [%]	4.5	41	32	45
Waist [cm]	68 – 102 83 (77; 95.8)	73 – 121 93 (89.8; 98.8)*	82 – 116 101 (95; 109.3)***	60 – 127 96.5 (89.5; 113.3)**
Systolic BP [mmHg]	107 – 140 121 (114.5; 127)	97 – 148 122 (110; 132)	102 – 158 120 (113; 126)	107 – 150 125 (112; 139)
Diastolic BP [mmHg]	66 – 90 78.5 (70.3; 81)	64 – 94 75 (69; 82)	60 – 91 73 (66; 81)	62 – 100 76 (67; 86)
Pulse [BPM]	64 – 96 83 (76.5; 88)	67 – 114 85 (79; 93)	61 – 112 89 (80; 94)	63 – 115 86 (79; 97)
Cholesterol [mmol/L]	4.16 – 6.03 4.96 (4.6; 5.54)	4.27 – 7.97 5.42 (4.6; 6.5)	4.6 – 9.1 6.24 (5.5; 7.1)***	4.47 – 8.78 6.01 (5.5; 6.4)**
Triglycerides [mmol/L]	0.88 – 2.09 1.2 (1.07; 1.32)	0.69 – 3.85 1.58 (1.21; 1.9)	0.84 – 3.68 2.3 (1.8; 3.1)***	0.58 – 3.81 2.3 (1.7; 3)***
HDL [mmol/L]	1.36 – 2.97 1.96 (1.8; 2.3)	1.22 – 3.07 1.8 (1.6; 2.2)	1.28 – 2.96 1.87 (1.6; 2.2)	1.08 – 2.97 1.73 (1.6; 2.2)
LDL [mmol/L]	1.69 – 7.93 2.35 (2.3; 2.8)	1.63 – 4.1 2.94 (2.3; 3.8)	1.49 – 5.76 3.3 (2.7; 4)**	1.78 – 4.8 3.03 (2.7; 3.7)*
nonHDL [mmol/L]	2.3 – 8.9 2.9 (2.7; 3.5)	2.0 – 5.8 3.5 (3; 4.1)	2.48 – 7.3 4.2 (3.7; 5)***	2.3 – 6.5 4 (3.6; 4.9)**
FPG [mmol/L]	3.7 – 5.1 4.25 (4.1; 4.5)	4.2 – 6.0 5.1 (4.9; 5.3)***	4.1 – 5.5 4.8 (4.5; 5)***	4.0 – 5.3 4.4 (4.2; 4.6)
C-peptide [pmol/L]	337.0 – 1262.0 658 (557; 888)	228.0 – 1619.0 681.5 (545; 961)	304.0 – 1716.0 696.5 (593; 880)	321.0 – 1705.0 600 (478; 798)
CP-RI [ng/mg]	3.45 – 14.47 6.65 (4.9; 9)	2.13 – 17.9 5.5 (4.1; 7.7)	2.2 – 13.43 6.17 (4.8; 7.1)	2.73 – 14.49 6.94 (5.6; 9.8)
HbA1c [% (mmol/mol)]	4.4 (25) – 5.3 (34) 4.9 (30) (4.7 (28); 5.1 (32.75))	4.5 (26) – 6.2 (44) 5.1 (32) (5.0 (31); 5.4 (35))**	4.4 (25) – 5.4 (36) 5.1 (32) (5.0 (31); 5.2 (33.25))	4.3 (24) – 5.7 (39) 5.1 (32) (4.8 (29); 5.2 (33))
Acetate [µmol/L]	0.97 – 23.05 9.42 (4.9; 13.8)	2.18 – 39.98 9.92 (5.5; 13)	1.99 – 42.69 9.4 (6; 14)	2.43 – 30.38 8.99 (5.14; 15)
Propionate [µmol/L]	0.01 – 1.62 0.78 (0.24; 1.3)	0.15 – 1.82 0.76 (0.4; 1.19)	0.11 – 2.48 0.75 (0.43; 1)	0.03 – 1.46 0.74 (0.38; 1.06)
Butyrate [µmol/L]	0.12 – 1.56 0.35 (0.22; 0.54)	0.12 – 1.07 0.31 (0.22; 0.4)	0.1 – 1.11 0.29 (0.19; 0.46)	0.11 – 0.6 0.32 (0.17; 0.45)
Valerate [µmol/L]	0.01 – 0.1 0.06 (0.033; 0.07)	0.01 – 0.12 0.05 (0.04; 0.07)	0.02 – 0.18 0.05 (0.03; 0.07)	0.03 – 0.3 0.05 (0.04; 0.08)
Hexanoate [µmol/L]	0.05 – 0.43 0.18 (0.13; 0.24)	0.08 – 0.43 0.17 (0.13; 0.23)	0.06 – 0.5 0.19 (0.13; 0.24)	0.09 – 0.43 0.2 (0.15; 0.24)
3-hydroxybutyrate [µmol/L]	16.32 – 55.79 31.84 (22.2; 42.9)	9.29 – 211.7 42.46 (25.8; 76.7)	12.8 – 196.1 43.83 (29.9; 74.6)*	27.7 – 299.4 91.2 (49.4; 137.2)***
2-hydroxybutyrate [µmol/L]	6.7 – 37.61 22.58 (15.9; 27.4)	11.3 – 69.76 23.17 (18.5; 28.2)	6.72 – 44.02 24.48 (18.4; 29.9)	14.43 – 71.31 26.12 (20.4; 38.1)
isobutyrate [µmol/L]	0.15 – 2.71 0.64 (0.35; 0.97)	0.14 – 1.54 0.72 (0.42; 0.99)	0.25 – 1.92 0.69 (0.42; 0.96)	0.2 – 1.32 0.56 (0.28; 0.93)
isovalerate [µmol/L]	0.23 – 0.73 0.37 (0.31; 0.41)	0.24 – 0.72 0.38 (0.32; 0.48)	0.1 – 0.69 0.33 (0.3; 0.43)	0.21 – 2.21 0.35 (0.3; 0.49)
2-methylbutyrate [µmol/L]	0.1 – 5.99 1.98 (0.64; 2.89)	0.15 – 15.31 1 (0.41; 3.48)	0.15 – 15.65 0.61 (0.36; 1.99)	0.27 – 52.05 1.23 (0.51; 2.66)
4-methylvalerate [µmol/L]	0.07 – 0.39 0.21 (0.12; 0.33)	0.06 – 0.66 0.21 (0.13; 0.31)	0.1 – 0.43 0.2 (0.17; 0.25)	0.14 – 0.5 0.26 (0.22; 0.32)

*p<0.05, **p<0.01, ***p<0.001 measured by Kruskal-Wallis test with Dunn´s post-hoc testing. The data are presented as medians with first and third quartiles in parentheses. FPG, fasting plasma glucose; oGTT, oral glucose tolerance test; BMI, body mass index; BP, blood pressure; HDL, high-density lipoprotein; LDL, low-density lipoprotein; HbA1c, glycated hemoglobin.

### Gut bacterial microbiota composition differs between pregnant women with and without diabetes in the first trimester

**Figure 1:**
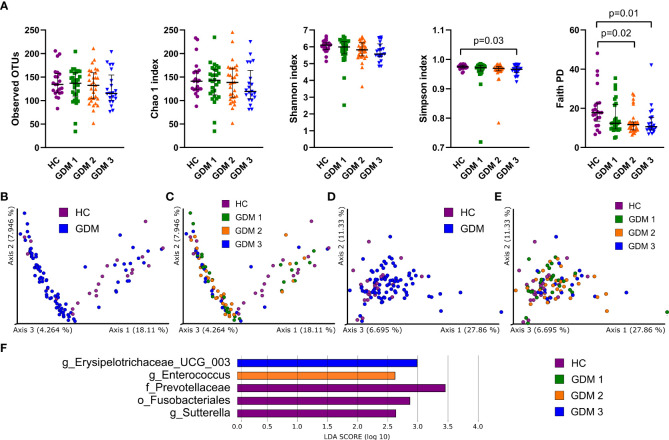
Gut bacterial community composition in the first trimester of pregnancy. **(A)** Alpha diversity indexes. Beta diversity of gut bacteria on unweighted **(B, C)** and weighted **(D, E)** UniFrac distance metric-based PCoA graphs show comparison of control healthy pregnant women (HC) with those with gestational diabetes mellitus (GDM) diagnosed at any time during the pregnancy. **(F)** LEfSe analysis of significantly different strains among groups. Statistically significant differences were measured by Kruskal-Wallis test with Dunn´s *post-hoc* testing. The data are presented as medians with interquartile range. OTUs, operational taxonomic units; PD, phylogenetic diversity.

To characterize differences in gut microbiota between pregnant women with and without diabetes, we collected fecal samples in the first trimester and processed them for sequencing analysis. We found mostly non-significant reduction in all alpha diversity indexes in the samples from women with GDM, except for the Simpson and Faith phylogenetic diversity indexes which describe the richness of the samples. Women diagnosed later during pregnancy either by FPG levels or by oGTT had significantly lower diversity compared to healthy controls (**Figure 1A**). We observed significantly different composition of gut microbiota in normoglycemic pregnant women and pregnant women with GDM measured by PERMANOVA based on unweighted and weighted UniFrac with values p=3x10^-3^ and p=6x10^-3^, respectively (**Figures 1B–E**). Subsequent LEfSe analysis identified bacteria significantly different among groups based on their relative abundances (**Figure 1F**). Gut microbiota of normoglycemic pregnancies was associated with increased abundance of family Prevotellaceae, order Fusobacteriales and genus *Sutterella*. The women who developed impaired insulin resistance later in pregnancy (GDM2) had higher abundance of genera *Enterococcus* and *Erysipelotrichaceae UCG-003*.

Prediction of metabolic pathways associated with the abundance of gut bacteria showed that most of them were linked to energy metabolism or active cell division, especially pathways producing components of cell membranes and cell walls (**Figure S1**).

### Gut fungal microbiota composition shows moderate changes between pregnant women with and without diabetes at the first trimester

**Figure 2:**
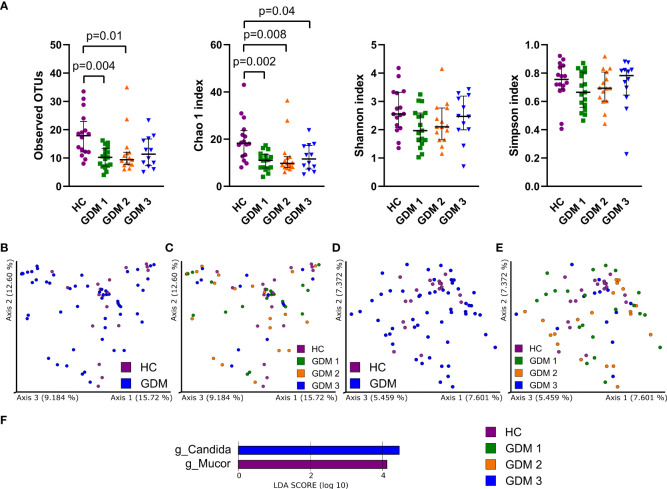
Gut fungal microbiota composition in the first trimester of pregnancy. **(A)** Alpha diversity indexes. Beta diversity of gut fungi on Bray-Curtis **(B, C)** and Jaccard **(D, E)** distance metric-based PCoA graphs show comparison of control healthy pregnancies (HC) with those with gestational diabetes mellitus (GDM) diagnosed at any time during the pregnancy. **(F)** LEfSe analysis of significantly different strains among groups. Statistically significant differences were measured by Kruskal-Wallis test with Dunn´s *post-hoc* testing. The data are presented as medians with interquartile range. OTUs,ndash; operational taxonomic units.

To characterize the gut mycobiota, we sequenced the ITS region in the samples from healthy pregnant women and pregnant women with GDM. Fungal alpha diversity indexes showed significant reduction in GDM pregnant women as described by species richness (observed OTUs) and by species abundance (Chao 1 index, **Figure 2A**). We did not observe any significant clustering of groups in neither Bray-Curtis dissimilarity plot based on fungal abundance nor in Jaccard distance plot comparing fungal composition among samples (**Figures 2B–E**). Using LEfSe analysis, we found that there was significant enrichment in genus *Mucor* in healthy pregnant women and that genus *Candida* was more abundant in the group of pregnant women with impaired oGTT in the third trimester (**Figure 2F**).

### GDM leads to different types of dysbiosis at the class level

**Figure 3:**
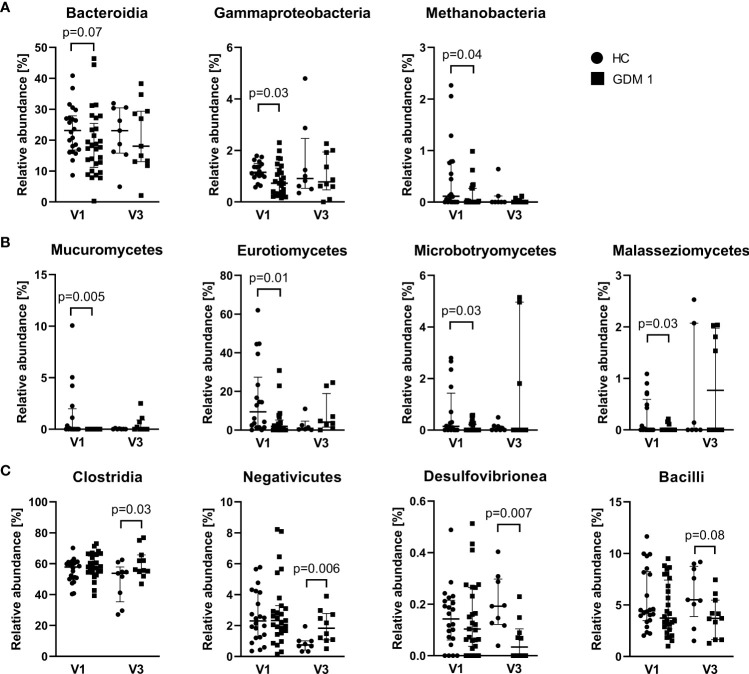
Differently abundant classes of bacteria and fungi between normoglycemic women (HC) and women with early-diagnosed GDM (GDM 1) at the first trimester – V1 **(A, B)** and the bacterial classes in the third trimester – V3 **(C)**. Only significantly different classes are shown. The data are presented as medians with interquartile range. The abundances were compared by Mann-Whitney U test and p < 0.05 was considered statistically significant.

Comparison of microbiota relative abundances in the samples collected during the first and third trimester showed significantly different patterns that distinguished healthy pregnant women and women with GDM. In the first trimester (V1), normoglycemic women were associated with higher abundance of bacterial classes Bacteroidia and γ-Proteobacteria, archeal class Methanobacteria, and fungal classes Mucuromycetes, Eurotiomycetes, Microbotryomycetes and Malasseziomycetes compared with pregnant women with GDM (**Figures 3A, B**). Interestingly, the differences in these classes were more or less narrowed later in the pregnancy. In the third trimester (V3), pregnant women with GDM showed significant increase in classes Negativicutes and Clostridia, especially of the family Oscillospiraceae, and lower abundance of classes Desulfovibrionea and Bacilli compared to normoglycemic women (**Figure 3C**). These two later classes included significantly more abundant genera *Bilophila*, *Leuconostoc*, *Streptococcus* and *Erysipelotrichaceae UCG-003* in healthy women (**Figure S2**). Although the analysis of fungal community showed no differences at class level during the third trimester, we found significant enrichment of family Debaryomycetaceae and genus *Rhodotorula* in women with GDM (**Figure S3**).

### Different intra- and inter-kingdom associations are linked to the GDM

Using Spearman correlation analysis, we found several significant associations among gut microbiota. Normoglycemic women (HC) showed a strong positive correlation of genera *Bacteroides* and *Roseburia* (r=0.75; p=6x10^-5^) and negative associations of genera *Dialister* with *Phascolarctobacterium* (r=-0.66; p=9x10^-4^) and *Parabacteriodes* with *Romboutsia* (r=-0.65; p=9x10^-4^) (**Figure S4**). Pregnant women with impaired FPG (GDM 1) had positive correlation of bacterial genus *Prevotella* with fungal *Cladosporium* (r=0.59; p=9x10^-4^) (**Figure S5**). Pregnant women with impaired FPG in the third trimester (GDM 2) showed strong negative correlations of genera *Dialister* with *Phascolarctobacterium* (r=-0.71; p=9x10^-6^) and *Holdemanella* with *Blautia* (r=-0.67; p=4x10^-5^). The GDM 2 group also showed several positive correlations, including the associations of genera *Fusicatenibacter* with *Agathobacter* (r=0.65; p=8x10^-5^), *Bifidobacterium* and *Collinsella* (r=0.64; p=10^-4^) and bacterial genus *Phascolarctobacterium* with archeal *Methanobrevibacter* (r=0.63; p=10^-4^) (**Figure S6**). Women with impaired oGTT in the third trimester (GDM 3) did not show any significant associations; the strongest one was negative correlation of bacterial genus *Holdemanella* with yeast *Candida* (r=0.62; p=4x10^-3^) (**Figure S7**).

### Correlation of bacterial strains with biochemical parameters and SCFA levels

**Figure 4:**
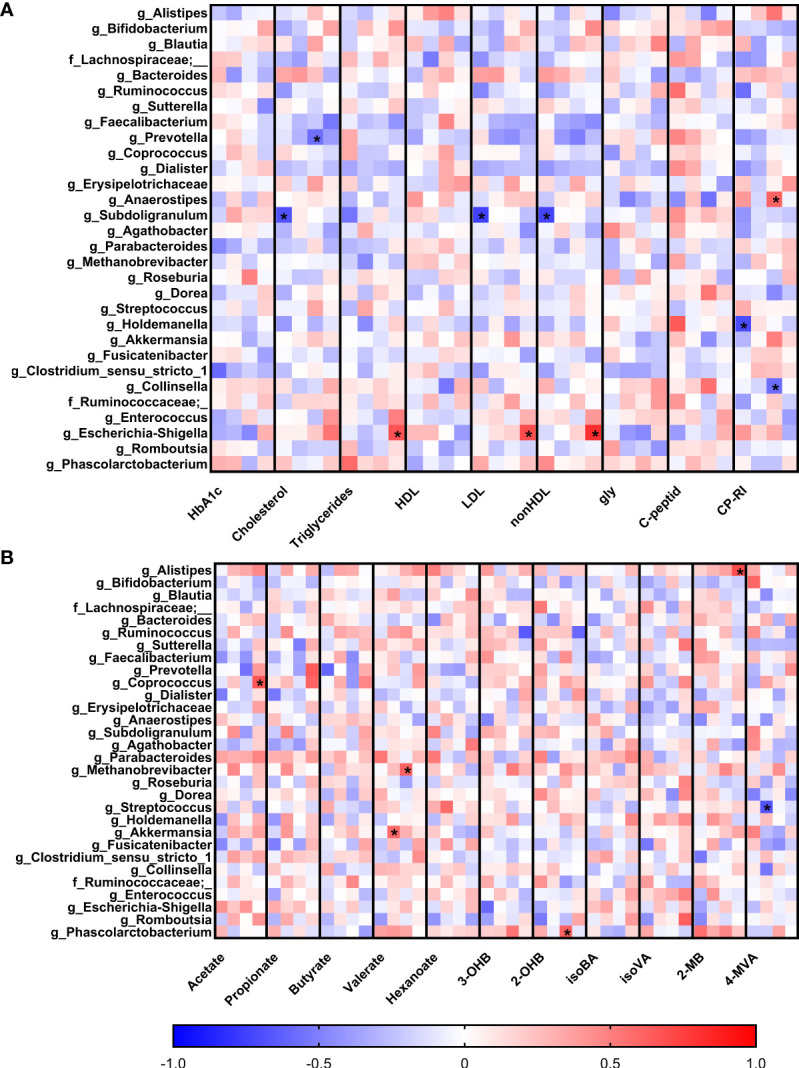
Correlations of serum biochemical parameters **(A)** and levels of short-chain fatty acids **(B)** with bacterial abundances. Within each column, the subcolumns are in order: healthy pregnant women, GDM1, GDM2 and GDM3. The strength and polarity of correlation is color-coded, e.g. negative correlation in shades of blue. All p-values were adjusted for multiple comparisons, p<0.001 was considered statistically significant and significant correlations were marked with the asterisks.

To observe early associations between bacteria and plasma parameters measured in the first trimester, Spearman correlation analysis was used (**Figure 4A**). In normoglycemic women, we found very strong negative correlation of genus *Subdoligranulum* with plasma levels of LDL (r=-0.75; p=9x10^-5^), nonHDL (r=-0.74; p=10^-4^) and cholesterol (r=-0.68; p=7x10^-4^) and genus *Holdemanella* which was also negatively associated with the level of CP-RI (r=-0.70; p=10^-3^). Interestingly, these correlations were not detected in early diagnosed group of pregnant women with GDM (GDM 1). The pregnant women with later onset of the GDM showed different associations, including negative correlation of genus *Prevotella* with cholesterol (r=-0.57; p=10^-3^) and genus *Collinsella* with CP-RI (r=-0.58; p=10^-3^) and positive correlation of genus *Anaerostipes* with CP-RI (r=0.61; p=5x10^-4^) and *Escherichia*/*Shigella* group with nonHDL (r=0.82; p=3x10^-6^), LDL (r=0.70; p=3x10^-4^) and triglycerides (r=0.67; p=6x10^-4^) levels.

Comparison of the associations of bacterial relative abundance with the levels of SCFA showed no specific pattern in normoglycemic women whereas GDM promoted some covariations (**Figure 4B**). In the GDM 1 group, genus *Akkermansia* positively correlated with the levels of valerate (r=0.58; p=10^-3^) and genus *Streptococcus* showed strong negative correlation with the levels of 4-methylvalerate (r=-0.67; p=8x10^-4^). In the GDM 2 group, archeal genus *Methanobrevibacter* positively correlated with the levels of valerate (r=0.61; p=4x10^-4^) and bacterial genus *Phascolarctobacterium* with 2-hydroxybutyrate levels (r=0.62; p=3x10^-4^). In the GDM 3 group, genera *Coprococcus* and *Alistipes* positively correlated with the levels of acetate (r=0.67; p=10^-3^) and 2-methylbutyrate (r=0.69; p=5x10^-4^), respectively.

## Discussion

In this study, we examined gut microbiome pattern of women in early stage of pregnancy to identify changes that are associated with GDM development. Systematic reviews have shown that although most of the studies observed an association between GDM and gut microbiota dysbiosis, no GDM-specific gut microbiota was identified (36, 37). Moreover, the contribution of gut mycobiome is often neglected. Though, there is a presumption that gut microbiota composition and function may contribute to the development of GDM (36). For this purpose, we focused on the composition of gut microbiota in early pregnancies.

In healthy population, gut microbiome contains six bacterial phyla: Firmicutes, Bacteroidetes, Proteobacteria, Actinobacteria, Fusobacteria, and Verrucomicrobia, with the dominance of the first two (38). Previous studies showed significant microbiota changes in normoglycemic women during pregnancy (20, 39, 40). Whether these changes contribute to or are a consequence of the development of GDM is a debated question. Several studies have reported increased abundance of Firmicutes or Actinobacteria and Proteobacteria in women with GDM (20, 21, 40, 41). In addition, enrichment of genera *Parabacteroides*, *Ruminococcus*, *Eubacterium*, *Prevotella*, *Collinsella*, *Rothia*, and *Desulfovibrio* has been also observed in pregnant women with GDM compared to normoglycemic controls (21, 42–44). On the contrary, increased abundance of Bacteroidetes and Actinobacteria as well as enrichment of *Faecalibacterium*, *Methanobrevibacter*, *Alistipes*, *Bifidobacterium* or *Eubacterium* has been described in normoglycemic pregnant women (40, 42, 43, 45). Most of these studies focused on the microbiota composition at the third trimester, i.e. after the onset of GDM. Therefore, we aimed more on the microbiota pattern in early pregnancy that could predict development of GDM. In our cohort of pregnant women, gut microbiota of normoglycemic women was associated with increased abundance of family Prevotellaceae, order Fusobacteriales and genus *Sutterella*. Interestingly, Wang *et al.* (2020) identified a significant decrease of the family Alcaligenaceae (including genus *Sutterella*) in the ascending colon of patients with T2DM. Subsequent experimental study showed increased abundance of *Sutterella* in the cecum of T2DM rats that underwent Roux-en-Y gastric bypass surgery (46). Thus, suggesting that this genus may beneficially affect glucose metabolism. Furthermore, we found that women who developed impaired insulin resistance later in pregnancy had higher abundance of genera *Enterococcus* or *Erysipelotrichaceae UCG-003*. This is in agreement with Ferrocino et al. (2018) who found that insulin resistance positively correlated with class Erysipelotrichia (45). Meanwhile, Crusell et al. (2018) observed reduction of *Erysipelotrichaceae* in women with GDM (21). Though, our results are supported by another study that found higher levels of *Erysipelotrichaceae* also in obese individuals (47). Since our study included obese individuals our results may be affected by this fact as well. Individuals with obesity have different profile of the gut microbiota in comparison to non-obese individuals (48). Moreover, obesity and GDM can influence many maternal and neonatal processes, includingthe breast milk microbiota and simultaneously the offspring gut microbiota. For example, compared to control samples, colostrum of women with either obesity or GDM was enriched in genera *Staphylococcus* or *Prevotella*, respectively (44).

Our study is one of the first to investigate the association between gut fungi and the GDM. Fungal communities in the gut constitute a minor component of the entire gut microbes thus are still poorly understood. According to recent shotgun metagenomic sequencing analysis, fungi represent approximately 0.1% of the total gut microbes (49). In our study, we found a significant enrichment of genus *Mucor* in healthy pregnant women. Members of this genus have been negatively correlated with obesity suggesting their association with microbiota of healthy lean individuals (50). Indeed, our cohort of normoglycemic pregnant women included only 4.5% of obese individuals. Recently, genus *Penicillium* has been associated with the gut mycobiota of healthy pregnant women (51) but we did not observe higher levels of this genus in our groups. In the group of pregnant women with impaired oGTT, we observed increased abundance of genus *Candida* in the third trimester. This is in agreement with very recent study by Ferrocino et al. (2022) who observed an increasing abundance of *Candida* between the second and third trimesters (52). *Candida albicans* inhabits the gastrointestinal tract, mouth and vaginal mucosa in 40 – 60% of healthy adults as a commensal organism, but it may cause disease in immunocompromised individuals (53, 54). Several studies have already reported increased abundance of *Candida albicans* in obese individuals and in patients with type 1 *diabetes mellitus* (T1DM) and T2DM (50, 55–57). Moreover, it is generally assumed that pregnant woman with GDM are more prone to *Candida* vaginal infection (58–60).

Decreased abundance of *Roseburia* and *Bacteroides* was observed in the GDM women compared to healthy pregnant women (42). In accordance, we determined positive correlation of these two bacteria in healthy women but not in the GDM group. In the groups of pregnant women with impaired FPG/oGTT in the third trimester, we found negative correlation of *Holdemanella* with *Blautia* and with yeast *Candida*, respectively. Romaní-Pérez et al. (2021) showed that *Holdemanella*, an intestinal bacterium isolated from metabolically healthy individuals, had anti-diabetic effect through glucagon-like peptide 1 signaling pathway and its supplementation improved glucose tolerance in a diet-induced obese mouse model (61). Increased abundance of *Collinsella* and reduced abundance of *Bifidobacterium* have been reported in pregnant women with GDM compared to healthy controls (21, 43). Nevertheless, we found positive correlation of *Collinsella* with *Bifidobacterium* in pregnant women with impaired FPG in the third trimester. In the same group, we also observed a positive correlation of *Methanobrevibacter smithii* and *Phascolartobacterium*. On the other hand, *Phascolartobacterium* negatively correlated with genus *Dialister* in the GDM2 and normoglycemic groups. Increased abundance of *Dialister* and reduced abundance of *Phascolartobacterium* have been related to impaired insulin sensitivity in obese individuals (62).

Healthy pregnancy is characterized by complex metabolic and hormonal changes. Plasma lipid concentrations change during pregnancy due to increasing insulin resistance. Serum levels of high-density lipoprotein-cholesterol (HDL-C), low-density lipoprotein-cholesterol (LDL-C), total cholesterol, and to lesser extent triglycerides (TG) are elevated throughout the pregnancy (63–65). In our study, we observed significantly higher levels of cholesterol, LDL-C, and TG in women with GDM compared to healthy women which is consistent with other studies (66–69). Moreover, it has been shown that gut microbiota can influence the levels of blood lipids (70, 71). Here, we found that lipid levels were linked to specific gut microbiota. In normoglycemic women, we found very strong negative correlation of genus *Subdoligranulum* with serum levels of LDL, nonHDL, and cholesterol. In contrast, in women with later onset of GDM, we found positive correlation of *Escherichia*/*Shigella* group with LDL, nonHDL, and triglycerides and negative correlation of genus *Prevotella* with total cholesterol. While the SCFA-producing genus *Subdoligranulum* has been connected with health promoting effects, the family Enterobacteriaceae has been enriched in GDM and has already been linked to T2DM and obesity (19, 43, 72).

In contrast to a well described role of lipids, the role of SCFA in pregnancy is still poorly understood. SCFA are derived from fermentation of carbohydrates and proteins by the gut microorganisms (73). They provide energy to colonocytes and maintain intestinal homeostasis by acting as signaling molecules that transmit messages between microbiota and host organs (74). We found positive correlations of valerate with genus *Akkermansia* and archaeon *Methanobrevibacter* in pregnant women with impaired FPG in the first and third trimester, respectively. In the GDM2 group, genus *Phascolarctobacterium* positively correlated with 2-hydroxybutyrate levels. In the study of Dudzik *et al.* (2017), an increase in 2-hydroxybutyrate in patients with diagnosed GDM in the second trimester of pregnancy was detected. Moreover, 2-hydroxybutyrate levels were significantly higher in GDM women that developed T2DM after parturition. Therefore, 2-hydroxybutyrate may serve as a prognostic tool for the prediction of early onset of the complications related to diabetes in women with GDM after delivery (75).

Overall, our study revealed significant differences in gut bacterial and fungal microbiota composition between healthy pregnant women and women who develop GDM in the first half of pregnancy. Furthermore, we identified correlations between individual microorganisms and plasma biochemical parameters, including SCFA levels. We found several microbial patterns that could be used in specific diagnostic test in the first trimester to identify women in higher risk of GDM. Nevertheless, our results need to be validated by further studies.

## Data availability statement

The datasets presented in this study can be found in online repositories. The names of the repository/repositories and accession number(s) can be found below: https://www.ncbi.nlm.nih.gov/bioproject/PRJNA833950.

## Ethics statement

The studies involving human participants were reviewed and approved by Ethics Committee at University Hospital Olomouc, Olomouc, Czechia (approval no. 120/17). The patients/participants provided their written informed consent to participate in this study.

## Author contributions

KK and DK designed the study. OK and DK collected clinical samples and biochemical data. NG and MV processed the samples for microbiota sequencing. MK processed sequencing data and bioinformatics. EI, DF and JF processed fatty acids analysis. MV, RR, ZZ, DF, MH and KK drafted the manuscript. All the authors read and approved the final version of the manuscript.

## Funding

This work was supported by the Ministry of Health of the Czech Republic (grant number NV18-01-00139), by Institute of Microbiology of the Czech Academy of Sciences Institutional Research Concept (RVO: 61388971), and by University Hospital Olomouc, MH CZ – DRO (FNOl, 00098892).

## Conflict of interest

The authors declare that the research was conducted in the absence of any commercial or financial relationships that could be construed as a potential conflict of interest.

## Publisher’s note

All claims expressed in this article are solely those of the authors and do not necessarily represent those of their affiliated organizations, or those of the publisher, the editors and the reviewers. Any product that may be evaluated in this article, or claim that may be made by its manufacturer, is not guaranteed or endorsed by the publisher.
