# Supplementary material for: Specific gut bacterial and fungal microbiota pattern in the first half of pregnancy is linked to the development of gestational diabetes mellitus in the cohort including obese women
Source: Front Endocrinol (Lausanne). 2022 Sep 5;13:970825. doi: 10.3389/fendo.2022.970825 (PMC9484836; doi:10.3389/fendo.2022.970825)
Supplement: Supplementary file 1 [file DataSheet_1.docx]

**Supplementary figures**

Manuscript: Specific gut bacterial and fungal microbiota pattern in the first half of pregnancy is linked to the development of gestational *diabetes mellitus* by Vavreckova et al.


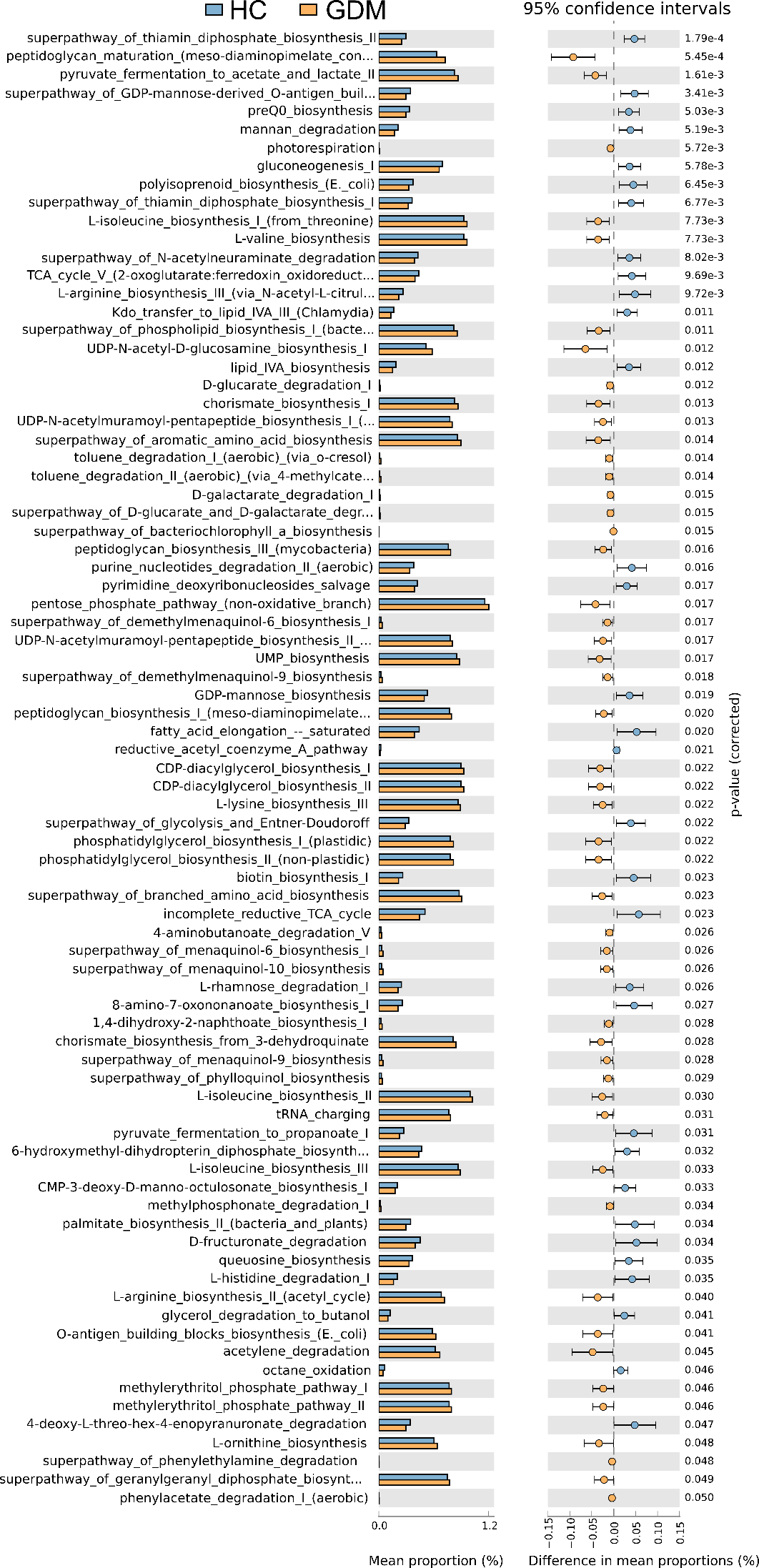


Figure S1 Functional analysis of gut microbiota. Metabolic pathways prediction based on the bacterial abundances was calculated by PICRUSt. Depicted are only pathways significantly different between normoglycemic women and women with GDM.


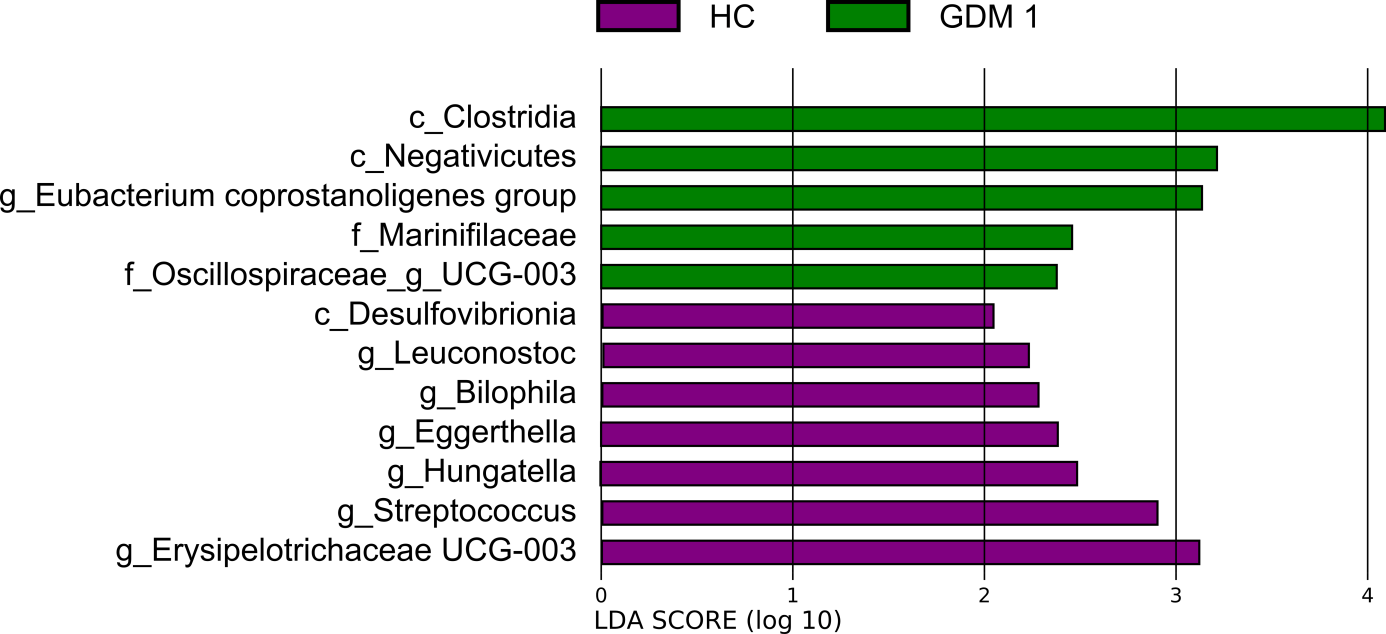


Figure S2 Significantly different bacterial strains between normoglycemic (HC) and diabetic (GDM 1) pregnancies based on the collection in the third trimester (V3). Determined by LEfSe analysis.


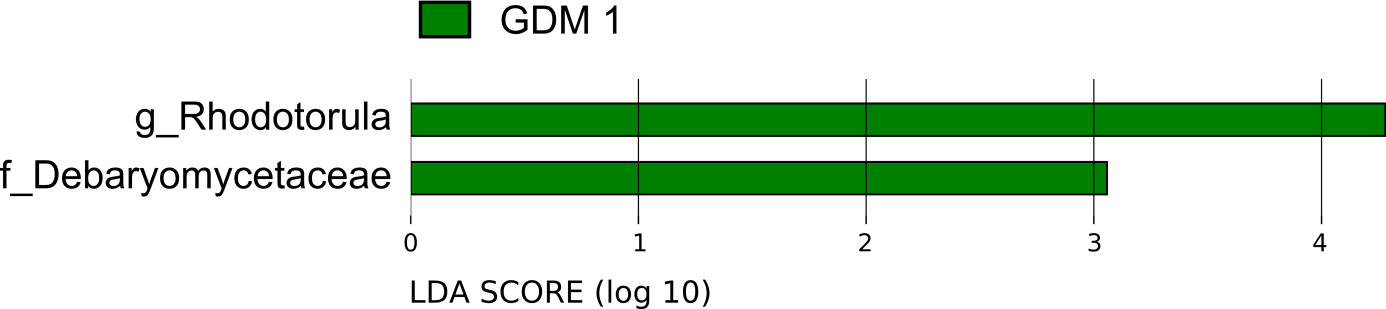


Figure S3 Significantly different fungal strains between normoglycemic (HC) and diabetic (GDM 1) pregnancies based on the collection in the third trimester (V3). Determined by LEfSe analysis.


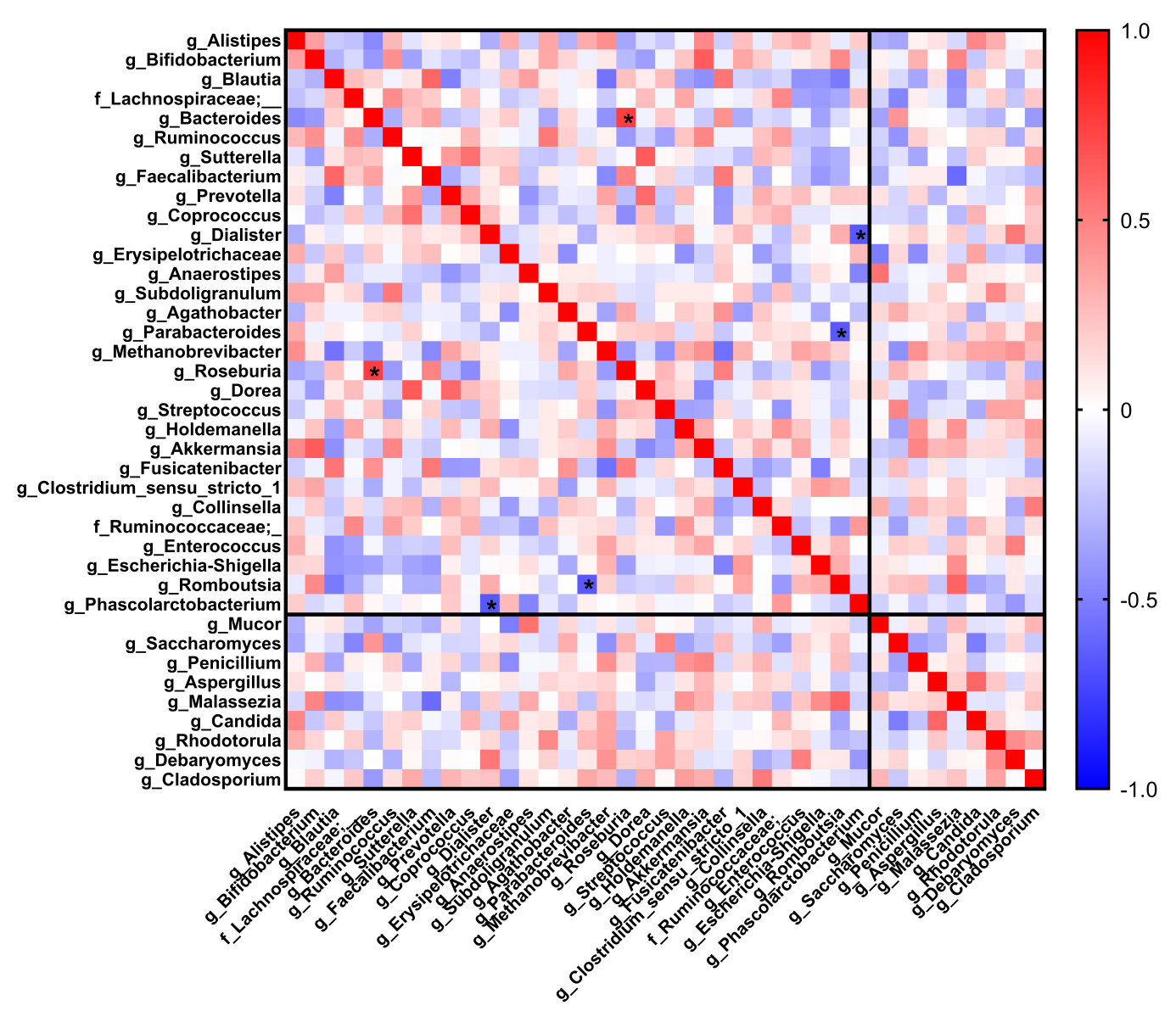


Figure S4 Inter- and intra-kingdom correlation of the gut microbiota in normoglycemic pregnancies. The strength and polarity of correlation is color-coded, e.g. negative correlation in shades of blue. All p-values were adjusted for multiple comparisons, p<0.001 was considered statistically significant and significant correlations were marked with the asterisks.


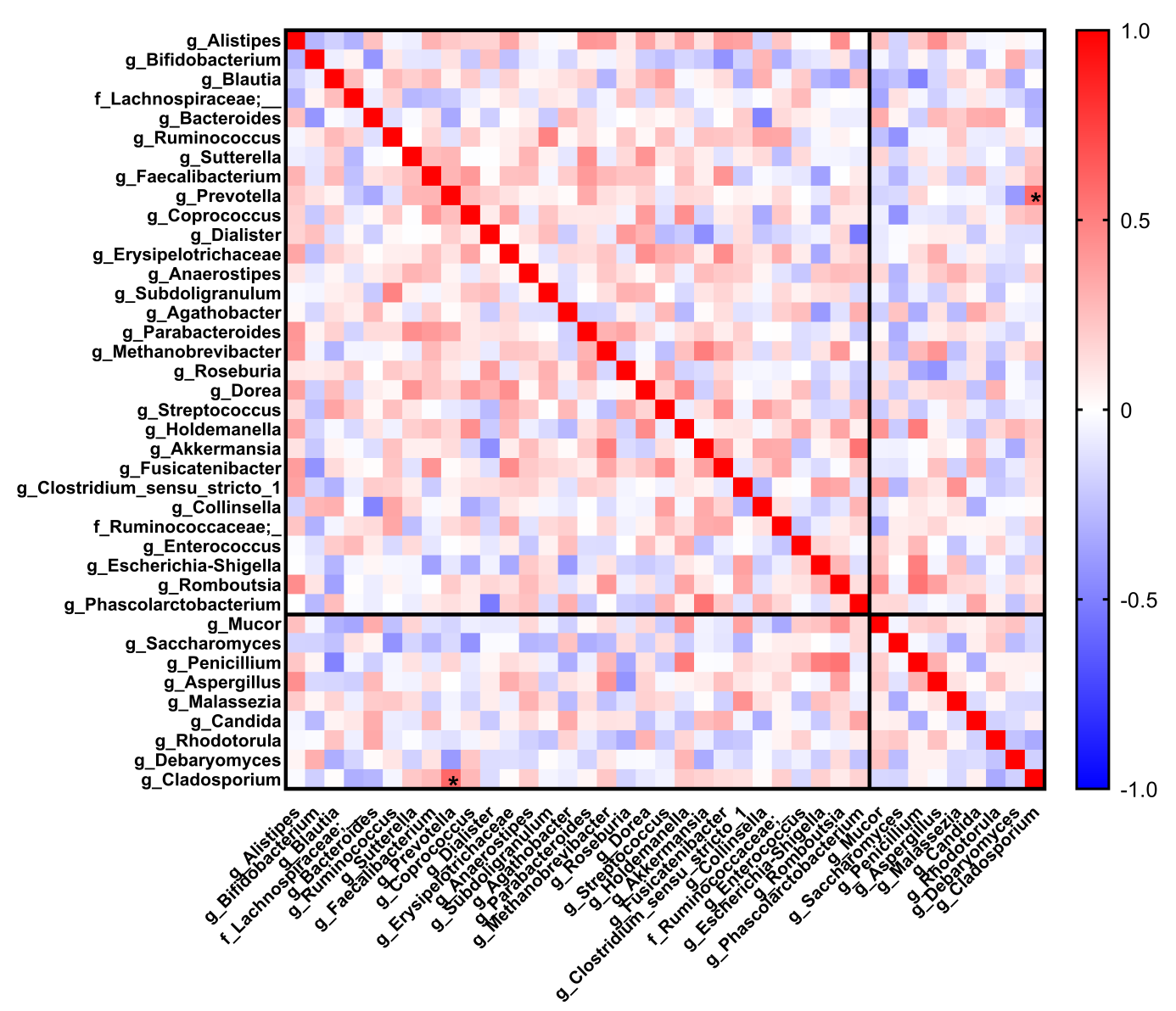


Figure S5 Inter- and intra-kingdom correlation of the gut microbiota in pregnancies with GDM diagnosed in the first trimester by impaired fasting plasma glucose levels (GDM 1). The strength and polarity of correlation is color-coded, e.g. negative correlation in shades of blue. All p-values were adjusted for multiple comparisons, p<0.001 was considered statistically significant and significant correlations were marked with the asterisks.


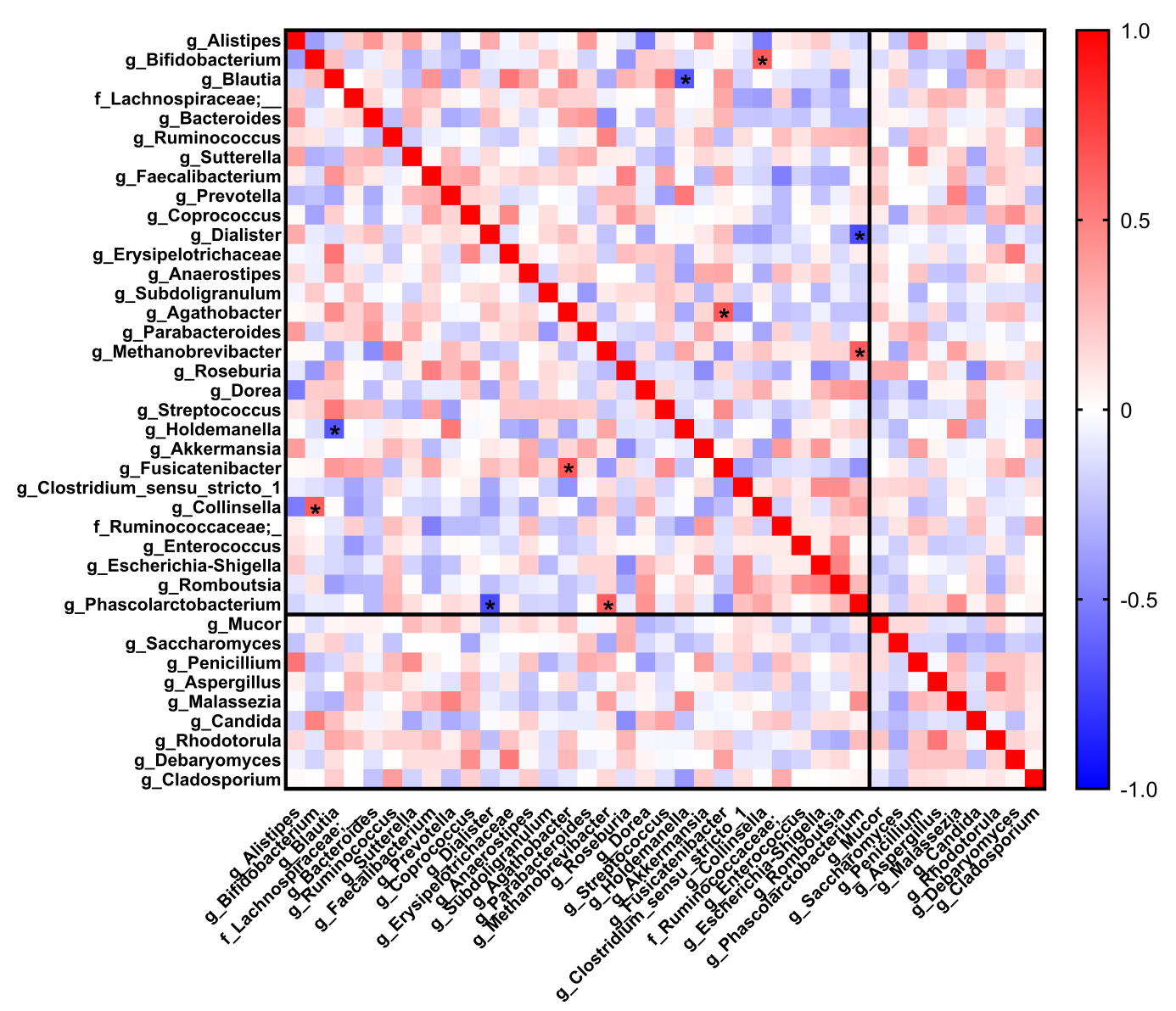


Figure S6 Inter- and intra-kingdom correlation of the gut microbiota in pregnancies with GDM diagnosed in the third trimester by impaired fasting plasma glucose levels (GDM 2). The strength and polarity of correlation is color-coded, e.g. negative correlation in shades of blue. All p-values were adjusted for multiple comparisons, p<0.001 was considered statistically significant and significant correlations were marked with the asterisks.


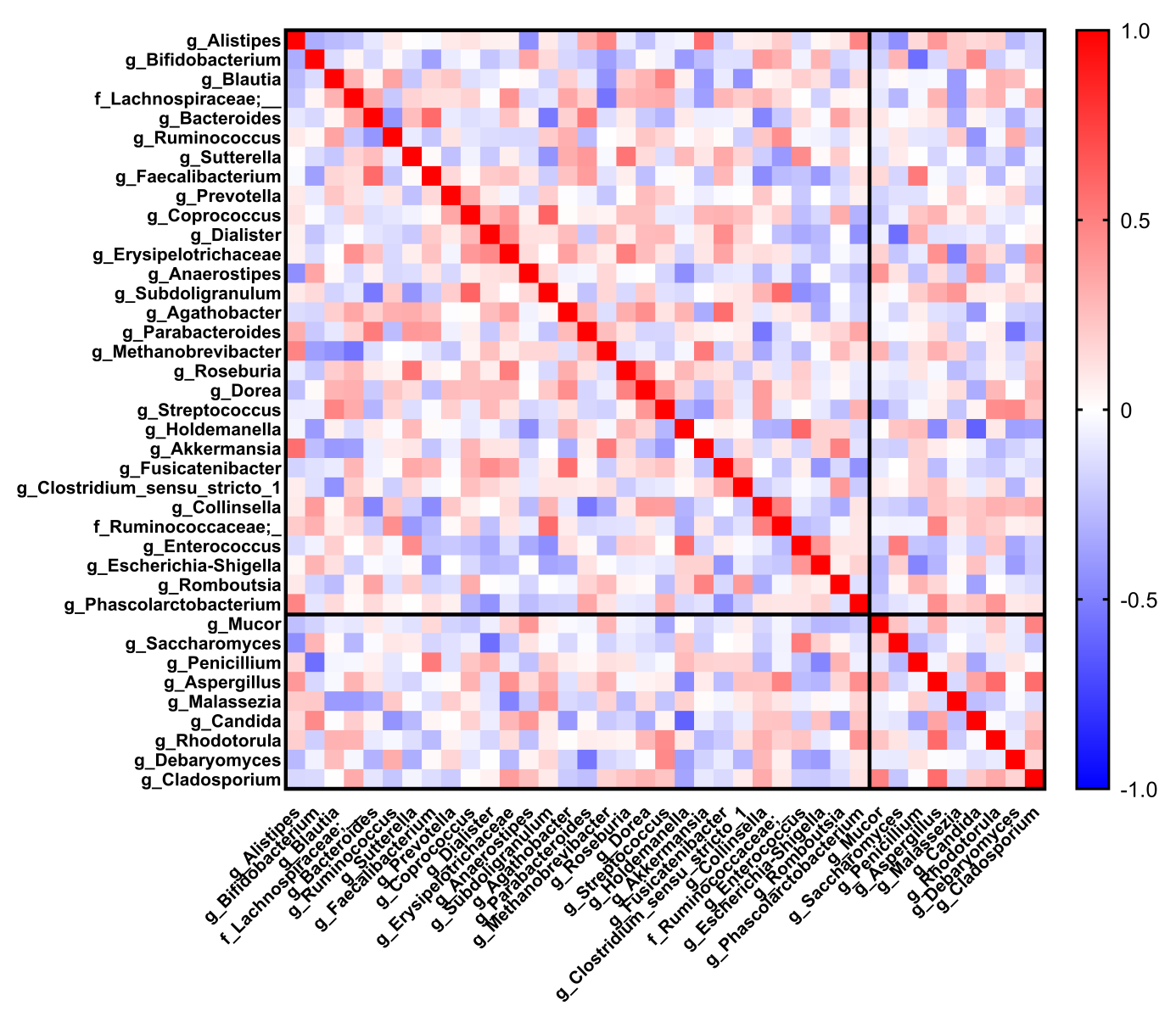


Figure S7 Inter- and intra-kingdom correlation of the gut microbiota in pregnancies with GDM diagnosed in the third trimester by impaired oral glucose tolerance test (GDM 3). The strength and polarity of correlation is color-coded, e.g. negative correlation in shades of blue. All p-values were adjusted for multiple comparisons, p<0.001 was considered statistically significant and significant correlations were marked with the asterisks.
